# Why and how we should join the shift from significance testing to estimation

**DOI:** 10.1111/jeb.14009

**Published:** 2022-05-18

**Authors:** Daniel Berner, Valentin Amrhein

**Affiliations:** ^1^ Department of Environmental Sciences, Zoology University of Basel Basel Switzerland

**Keywords:** compatibility interval, effect size, null hypothesis, *p*‐value, scientific method, statistical inference

## Abstract

A paradigm shift away from null hypothesis significance testing seems in progress. Based on simulations, we illustrate some of the underlying motivations. First, *p*‐values vary strongly from study to study, hence dichotomous inference using significance thresholds is usually unjustified. Second, ‘statistically significant’ results have overestimated effect sizes, a bias declining with increasing statistical power. Third, ‘statistically non‐significant’ results have underestimated effect sizes, and this bias gets stronger with higher statistical power. Fourth, the tested statistical hypotheses usually lack biological justification and are often uninformative. Despite these problems, a screen of 48 papers from the 2020 volume of the Journal of Evolutionary Biology exemplifies that significance testing is still used almost universally in evolutionary biology. All screened studies tested default null hypotheses of zero effect with the default significance threshold of *p* = 0.05, none presented a pre‐specified alternative hypothesis, pre‐study power calculation and the probability of ‘false negatives’ (beta error rate). The results sections of the papers presented 49 significance tests on average (median 23, range 0–390). Of 41 studies that contained verbal descriptions of a ‘statistically non‐significant’ result, 26 (63%) falsely claimed the absence of an effect. We conclude that studies in ecology and evolutionary biology are mostly exploratory and descriptive. We should thus shift from claiming to ‘test’ specific hypotheses statistically to describing and discussing many hypotheses (possible true effect sizes) that are most compatible with our data, given our statistical model. We already have the means for doing so, because we routinely present compatibility (‘confidence’) intervals covering these hypotheses.

## Graphical abstract

Inference in ecology and evolution is still mostly based on significance testing. We summarize problems with this approach and argue that it should be replaced by the adequate description of effect size estimates.

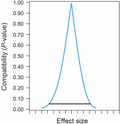

## 1 INTRODUCTION

In 2019, the editors of a special issue of The American Statistician on ‘Statistical inference in the 21st century’ concluded ‘that it is time to stop using the term “statistically significant” entirely’ (Wasserstein et al., 2019). More than 800 scientists subscribed to a commentary titled ‘Retire statistical significance’ (Amrhein et al., 2019a). Some biologists now claim that ‘the reign of the *p*‐value is over’ (Halsey, 2019) and that ‘it is time to move away from the cult around binary decision making and statistical significance’ (Muff et al., 2022), while numerous scientific journals publish editorials or revise their guidelines, asking their authors to diminish the importance attributed to null hypothesis significance testing (e.g. Davidson, 2019; Harrington et al., 2019; Krausman & Cox, 2019; Michel et al., 2020).

Already in the middle of the last century, scientists like Yates (1951) and Rozeboom (1960) called for shifting attention from significance testing to estimation. Yates and Healy (1964) asked for a reform in the teaching of statistics, noting that significance tests ‘are popular with non‐statisticians, who like to feel certainty where no certainty exists’ and that ‘it is depressing to find how much good biological work is in danger of being wasted through incompetent and misleading analysis of the numerical results’. After decades of heated discussions about a methodological approach deeply ingrained in our scientific culture (reviewed in Amrhein et al., 2017; Gigerenzer, 2018; Hurlbert & Lombardi, 2009; Johnson, 1999; Mayo, 2018; Oakes, 1986; Szucs & Ioannidis, 2017; Ziliak & McCloskey, 2008), a paradigm shift seems finally under way. Even in the most selective journals, it is now possible to publish papers using traditional frequentist methods without any reference to *p*‐value thresholds and statistical significance (e.g. Senzaki et al., 2020).

In this note, however, we report that this development has so far been largely ignored by evolutionary biologists, for example by the authors of 48 papers that we randomly selected from the 2020 volume of the Journal of Evolutionary Biology. We therefore provide a summary of the main problems with the traditional culture of analysing, presenting and interpreting scientific data based on statistical significance. We then make recommendations how we can participate in the paradigm shift and contribute to improving scientific practice by using a more nuanced form of statistical inference.

## 2 WHAT ARE THE PROBLEMS?

As in many fields of research, a study in ecology and evolutionary biology typically starts with observational or experimental data acquired because we suspect a relationship between variables (for simplicity, we use the terms ‘relationship’ and ‘effect’ interchangeably). For statistical analysis, the most popular approach seems to be hypothesis testing. According to the methods developed by Jerzy Neyman and Egon Pearson, this would require pre‐analysis specification and justification of the tested (null) hypothesis, of an alternative hypothesis and of decision rules (Goodman, 2016; Greenland, 2021; Lehmann, 2011).

If following this procedure, our aim is to ‘reject’ or ‘accept’ hypotheses, a minimum requirement would be to make defensible choices of alpha *and* beta error probabilities (i.e. the probabilities of rejecting the null hypothesis if in reality it is true [‘false positive’], and of failing to reject the null hypothesis if it is false [‘false negative’]), as well as calculating statistical power (the probability of rejecting the null hypothesis if it is false) before the data for the study are collected. ‘Defensible choices’ means that acceptable error probabilities are set by taking into account the costs and implications of committing the above errors within the research context of our study (Greenland, 2017).

In practice, however, data in ecology and evolutionary biology are typically collected without any pre‐study determination and justification of reasonable null and alternative hypotheses or of decision rules and decision costs (see our survey below, and Anderson et al., 2021 for power analysis in ecology). Instead, the data are subjected to null hypothesis significance testing (NHST) with a default null hypothesis of a zero relationship and a default *p*‐value threshold (accepted alpha error rate, or alpha level) of *p* = 0.05. Also, in the absence of pre‐study power calculation, there is no information on the beta error rate, which is 1‐power. The test thus yields a *p*‐value reflecting the probability of observing a relationship at least as large as the one we found, given that the null hypothesis of ‘no relationship’ is true—and given that all other assumptions about the test and about the entire study are correct (Amrhein et al., 2019b).

If the *p*‐value is below 0.05, we usually interpret this as indirect evidence against the null hypothesis, thus drawing the reverse conclusion that the null hypothesis is unlikely given our data. Hence, we reject the null hypothesis and infer that a relationship exists—the test was ‘statistically significant’. If the *p*‐value is equal to or greater than 0.05, we are inclined to say that no relationship exists, or at least that we were not able to demonstrate it; the test was ‘statistically non‐significant’. An equivalent approach is evaluating whether a 95% confidence interval overlaps the null hypothesis of zero effect, in which case the null hypothesis would not be rejected.

This standard protocol of NHST with unjustified alpha and unknown beta error probabilities discredits the originally intended rationale of Neyman–Pearson hypothesis tests (Szucs & Ioannidis, 2017), and the associated dichotomous inference about the presence or absence of a relationship rests on several misconceptions. We now discuss four of these misconceptions that seem to be the most important to us.

### 2.1 Misconception 1: The *p*‐values emerging from our analyses are reliable

**FIGURE 1:**
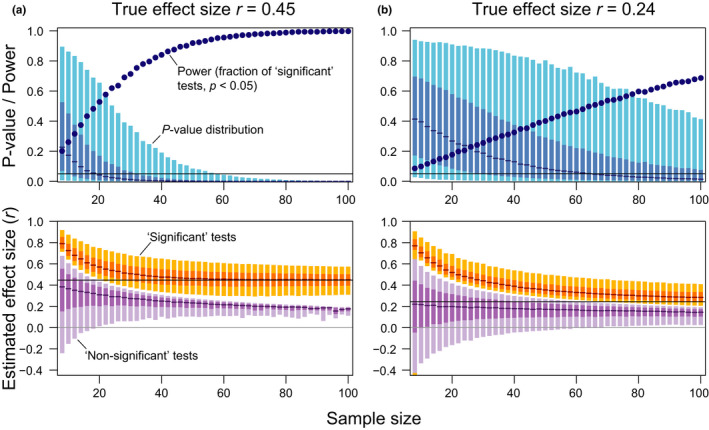
*P*‐values and effect sizes in statistical hypothesis tests in relation to sample size. Shown are summary statistics based on null hypothesis significance tests of a simulated true correlation between a predictor and a response variable, for sample sizes ranging from eight to 100 in increments of two. The simulated true effect sizes are Pearson correlation coefficients that were chosen to be relatively large (*r* = 0.45) in (a) and smaller (*r* = 0.24) in (b). For each sample size, 10,000 replicate bivariate data sets were simulated and tested. In the upper panels, the blue bars represent the central 90% (light blue) and 50% (darker blue) of the *p*‐value distribution among the replicate tests, and the short horizontal lines are the medians. The dark blue bullet points indicate power, which is the fraction of tests that are ‘statistically significant’ (*p* < 0.05; significance threshold shown as black horizontal line). For the same set of simulations, the lower panels visualize the distribution of the effect size estimates, which are the correlation coefficients (*r*) observed in the samples. The effect sizes are presented separately for the subset of replicate tests that were ‘statistically significant’ (orange) or ‘statistically non‐significant’ (purple). The light and darker bars represent the central 90% and 50% of the effect sizes observed across the replicate tests, and the short horizontal lines are the medians. The true effect sizes underlying the simulations are shown as black horizontal lines. Note that in (b), the 90% interval for the significant tests with *n* = 8 extended to −0.75 but was truncated to facilitate presentation; with small sample size, the effect size distribution of significant tests was bimodal because a fraction of the significant tests had strong negative correlations. Overall, the simulations highlight that when the true effect size and/or the sample size is modest, *p*‐values are highly variable, and most ‘statistically significant’ effect estimates are biased upwards. Analogously, ‘statistically non‐significant’ effect estimates tend to underestimate the true effect size, and here the bias gets stronger with increasing sample size and/or when the true effect size is substantial

*P*‐values are contingent on the sample data obtained and hence represent random variables themselves. They are expected to vary from replication to replication of a study, even for surprisingly large sample sizes (Cumming, 2014; Halsey et al., 2015). This is illustrated in Figure 1, based on simulations of correlations between two variables (methodological detail is given in Appendix S1, and the simulation code written in the R language (R Core Team, 2020) is available from the Dryad repository). For a true correlation of *r *= 0.45, arguably qualifying as a substantial effect size in the field of ecology and evolution (Møller & Jennions, 2002), *p*‐values are highly variable with sample sizes up to around *n* = 20 to 30 (Figure 1a). When the true effect size is smaller (*r* = 0.24), *p*‐values span a remarkably wide range even when sample sizes approach *n* = 100 (Figure 1b).

This variability of the *p*‐value is impressive enough in simulations in which the true properties of the data generating mechanism are known and all assumptions underlying our statistical model are met (because we simulated the data according to this model). In reality, however, model assumptions will almost always be violated to some degree (Higgs, 2019). Furthermore, we often do not discuss or are not even aware of all assumptions (Amrhein et al., 2019b). Departures from model assumptions, however, invalidate *p*‐values and other statistical measures at least to some degree. This becomes obvious if the assumption of ‘no *p*‐hacking’ is violated, in which case the reported *p*‐values are close to worthless. No *p*‐hacking means that ‘analytical decisions were taken independently from the obtained data and would have been the same given other possible data’ (Gelman & Loken, 2014)—an assumption that is probably almost always violated to some degree, albeit often unknowingly and with the best of intentions.

Given all the random noise (stochastic variability as shown in Figure 1) and non‐random noise (assumption violations, unaccounted methodological or biological differences among studies), it is not surprising that meta‐analyses (Gurevitch et al., 2018; Halsey, 2019) and large‐scale replication projects (Errington et al., 2021; Open Science Collaboration, 2015) reveal dramatic variability in *p*‐values from study to study. This variability is not a problem of the *p*‐value by itself, but simply reflects variation in the data and analytical decisions from study to study. However, if *p*‐values are used with a threshold for dichotomous judgments about the ‘presence’ or ‘absence’ of an effect, or about whether an effect is ‘real’ or not, as is typical within the NHST framework, we may easily reach overconfident conclusions in either direction. Such overconfident dichotomous generalizations from single studies often lead to the erroneous perception that replication studies show ‘conflicting’ evidence and that science is in a general replication crisis (Amaral & Neves, 2021; Amrhein et al., 2019a, b).

Another issue is that many studies in ecology and evolution report dozens if not hundreds of *p*‐values, and often many more *p*‐values are calculated but not reported (Fraser et al., 2018). By definition, some proportion (depending on the adopted significance threshold) of these tests must turn out ‘statistically significant’ even if the tested (null) hypothesis is true. This multiple comparison problem is probably widely known in principle, but routinely ignored when drawing conclusions about analytical results. Moreover, possible strategies to adjust for multiple comparisons are debated and there are no easy solutions (Greenland, 2021). The inconvenient message is that conclusions drawn from individual *p*‐values become more unreliable the more *p*‐values are calculated. It is therefore particularly poor practice to present just a subset of the calculated *p*‐values chosen for their significance while hiding the rest; it is equally poor practice to calculate *p*‐values only on subsets of data that look most promising after plotting them. Complete reporting is crucial, even if it may appear embarrassing to present numerous *p*‐values and plots in a paper or appendix.

Taken together, while still often perceived as the centrepiece of a statistical analysis suited for dichotomous decision making, *p*‐values are generally no more than crude indicators of how ‘compatible’ a statistical model is with our observed data, given that all assumptions are correct (Amrhein et al., 2019b; Bayarri & Berger, 2000; Greenland, 2019; Rafi & Greenland, 2020; Wasserstein & Lazar, 2016). One of these assumptions is that our tested (null) hypothesis is true. A small *p*‐value then suggests that at least one of the assumptions is violated; whether and to what degree this can be interpreted as ‘evidence against the null hypothesis’ (Amrhein & Greenland, 2022; Hartig & Barraquand, 2022; Muff et al., 2022) is often so uncertain that we should refrain from making dichotomous decisions based on single studies (Amrhein et al., 2019b). This is one of the reasons why we should reduce the importance we assign to isolated studies for drawing conclusions and making decisions (Amaral & Neves, 2021; Nelder, 1986; Nichols et al., 2019, 2021).

### 2.2 Misconception 2: Statistical non‐significance indicates the absence of an effect

It has been known for more than a century that the absence of statistically significant evidence is not evidence of absence (Altman & Bland, 1995; Fisher, 1935; Pearson, 1906). Yet, the wrong conclusion of ‘no effect’ because *p* > 0.05 is still drawn in around half of the published papers across multiple research fields (Amrhein et al., 2019a).

Within the Neyman–Pearson hypothesis testing framework, we may ‘accept’ a null hypothesis if *p* > alpha and behave as though it were true if we know, approximately, how often we are in error when making that decision (given that all model assumptions are correct). However, since we usually do not formally calculate statistical power in the planning stage of a study, we usually have no idea how often we would commit the beta error of falsely accepting a wrong null hypothesis (because beta = 1‐power). This also means that the significance threshold (alpha level) of *p* = 0.05 is not ‘a compromise between alpha and beta error rates’, as one can hear or read sometimes (e.g. Hartig & Barraquand, 2022); to reach such a compromise, one would have to consider the power of the test as well as the costs of making alpha and beta errors in the context of the study.

If we calculated power for our studies in ecology and evolution that typically have small effect sizes (Møller & Jennions, 2002), we would likely find that our beta error probability is high: Across 44 reviews in the social, behavioural and biological sciences, average power to detect such effects was merely 24%, and hence, the average beta error probability was 100–24 = 76% (Smaldino & McElreath, 2016; see also Button et al., 2013; Jennions & Møller, 2003).

Even with the widely recommended power of 80%, the probability of falsely accepting a wrong null hypothesis (beta = 20%) would be four times the probability of falsely rejecting a true null hypothesis (alpha, by default set to 5%). This reveals another oddity of the current application of hypothesis tests: why should a four times higher beta error rate be tolerable across scientific disciplines and different research contexts within disciplines, implying that it is generally four times less costly to wrongly claim ‘there is no relationship’ than to wrongly claim ‘there is a relationship’? As is known since hypothesis tests were invented, false negatives can be more costly than false positives, depending on the subject and purpose of a study. For an endangered species, for instance, a false‐negative inference (e.g., failing to recognize a population decline, although it is taking place, which may result in extinction) can cause more harm than a false‐positive (wrongly claiming a population decline, which may stimulate more research or lead to conservation measures that may not be strictly necessary).

Low statistical power of our research and high beta error probabilities are thus one of the reasons why claims of ‘no relationship’ are usually unwarranted. For illustration, consider the substantial true correlation between two variables shown in Figure 1a. Using a sample size of 20, we obtain a ‘statistically non‐significant’ result in roughly half of the tests, hence inferring ‘no relationship’ would be erroneous in half of the studies; and when the true correlation is weaker (Figure 1b), we would be wrong in half of the cases even for sample sizes beyond 60.

**FIGURE 2:**
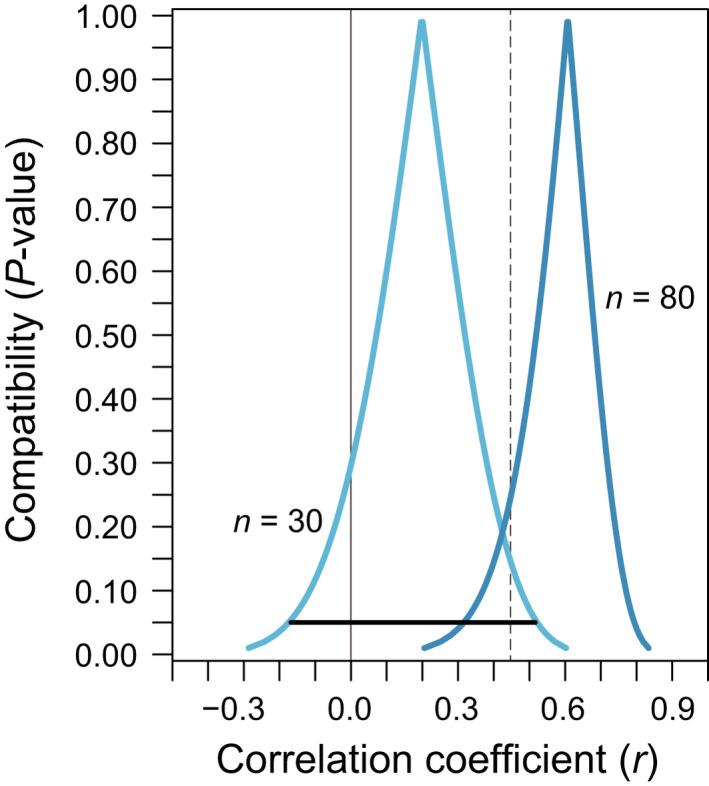
Visualizing the range of values for the true effect size (or in other words, of hypotheses) that are most compatible with the observed data, given the statistical model, by means of compatibility curves. The two curves illustrate the most compatible values for the true Pearson correlation coefficients based on two exemplary simulated samples of *n* = 30 and *n* = 80, generated using the bivariate simulation model underlying Figure 1a. Unlike in real research, the true correlation coefficient is known to be *r* = 0.45 (dashed vertical line). The black horizontal line under the left curve shows the 95% compatibility (‘confidence’) interval based on the *n* = 30 sample. Here, one of the many values that are most compatible is a zero relationship (solid vertical line). Because zero is included, this interval would traditionally be called ‘statistically non‐significant’, although zero is clearly not the value most compatible with the data: zero is not at the highest point of the compatibility curve. One can imagine the compatibility curve as horizontally stacked compatibility intervals, with compatibility levels ranging from near zero to one; from the bottom, the lowest interval is approximately the 100%‐interval and the highest is the 0%‐interval. The peak of the curve is thus the shortest (0%) compatibility interval that is just one point, known as the point estimate. This point estimate, which is the observed effect size, is the correlation coefficient estimate that is most (100%) compatible with the sample data and the statistical model (but because many other hypotheses are also reasonably compatible, 100% compatibility does not imply truth). The curve was drawn by determining the stacked compatibility intervals non‐parametrically based on quantiles from a distribution obtained by bootstrapping the original samples and recalculating the correlation coefficient 100,000 times, but a similar curve would arise when stacking conventional parametric ‘confidence’ intervals (see Appendix S1). Another way to interpret the compatibility curve is that it indicates the *p*‐values one would obtain, given the sample data and the statistical model, when using a correlation coefficient given on the x‐axis as null hypothesis in a test. The 95% interval shown therefore covers correlation coefficients that have *p* > 0.05 and are thus most compatible with the data and the model. For more details on the interpretation of compatibility curves, see section 4.3 of this paper as well as Infanger and Schmidt‐Trucksäss (2019), Poole (1987), and Rafi and Greenland (2020)

However, even with high statistical power, a large *p*‐value does not mean that the null hypothesis of a zero effect can be considered true (Greenland, 2012), because many other hypotheses are probably as compatible or more compatible with the data. This becomes obvious by imagining a ‘non‐significant’ confidence interval overlapping the null hypothesis of a zero relationship. A 95% confidence interval shows not just one, but all the values (hypotheses, possible values for the true effect size, or possible parameter values) that would, if used as null hypotheses, produce *p* > 0.05 and would thus not be rejected when tested using our data (Amrhein et al., 2019b; Cox & Hinkley, 1974; Greenland et al., 2016; Rafi & Greenland, 2020). In a ‘non‐significant’ interval, the hypothesis of ‘zero relationship’ would not be rejected and is thus reasonably compatible with our data—but all the other values covered by the interval are also reasonably compatible with our data; and usually the values near the point estimate are more compatible with the data than a value of zero effect (see section 4.3 and Figure 2).

Often, an interval covering the null value will also cover values of scientific or practical importance. Only if all the values inside an interval seem unimportant within a given research context and are thus of practical equivalence to the null, it may be justified to conclude that the study results indicated no effects of practical importance (Amrhein et al., 2019a, b; Colegrave & Ruxton, 2003; Hawkins & Samuels, 2021).

### 2.3 Misconception 3: Statistically significant effect sizes are reliable

Unless the power of a hypothesis test is near one, a significant test result will, on average, be associated with an overestimated (inflated) effect size. The reason is that due to sampling variation, some studies will find an effect that is larger than the true population effect size; and those studies are more likely to be significant than studies that happen to find smaller, or more realistic, effects. The lower the statistical power, the more exaggerated a relationship needs to be to become statistically significant, and thus the stronger the overestimation of significant effect sizes (Colquhoun, 2014; Gelman & Carlin, 2014; van Zwet & Cator, 2021). Since actual power of studies is usually much lower than the power calculated in the planning stage of a study (because the true effect sizes are usually smaller than the effect estimates used in those power calculations), the significant effects from even the most carefully planned experiments are likely to show exaggerated effect estimates (van Zwet et al., 2021).

In our correlation example based on the stronger relationship (*r *= 0.45), simulated replications capturing an effect equal to or smaller than the true magnitude essentially cannot produce a significant test result unless the sample size is greater than about *n* = 20 (Figure 1a; in other words, with *n* <= 20, the 90% intervals of effect size estimates of significant studies cover only magnitudes greater than the true value). With *n* = 20, the median observed correlation in significant tests overestimates the true correlation by 27%, and sample sizes of at least *n* = 50 or 60 are needed to achieve reasonably accurate significant effect size estimates in our simulation example. When the true effect size is smaller (*r *= 0.24), the median effect size estimates of significant tests remain biased upwards by at least 16% even when sample sizes approach *n* = 100 (Figure 1b).

Analogously, effect sizes observed in non‐significant tests tend to underestimate the magnitude of true effects (Figure 1). Perhaps somewhat counterintuitively, this downward bias becomes stronger with larger sample size or with a larger true effect size; the reason is that with high statistical power, most tests on a true effect will turn out significant, and only studies with extreme underestimates will be non‐significant. This bias may play a minor role in practice, since with high statistical power, non‐significant tests should be rare (Figure 1a); however, whenever we focus on results because they are non‐significant, our effect estimate will be more misleading the higher our statistical power is.

In summary, the usual filtering of results based on statistical significance causes systematic overestimation of effect sizes in our studies, as well as in reviews and news based on those studies. This bias can be reduced by publishing and discussing all results, with a focus on describing interval estimates rather than on claiming ‘statistical significance’ or ‘non‐significance’. Accordingly, in pre‐registered replication studies publishing all results irrespective of their *p*‐values, effect sizes are usually substantially smaller than in the original studies that likely filtered results by statistical significance to decide what is reported and discussed. For example, in a project replicating 50 experiments from preclinical cancer biology, the median effect size across the replications was only 1/7 of the median effect size in the original experiments (for original positive results; Errington et al., 2021).

### 2.4 Misconception 4: Our tests evaluate meaningful hypotheses

NHST can be understood as a vacuous ritual established to give us the feeling that our judgment about observed effects is reliable and objective, hence scientific (Gigerenzer, 2018; Gigerenzer & Marewski, 2015; Yates & Healy, 1964). This becomes evident when considering the hypotheses actually tested. The default hypothesis evaluated is a point null hypothesis of ‘zero relationship’, yet we initiated our research because in the light of pre‐existing evidence, or at least of intuition or wishful thinking, we suspected that a non‐zero relationship in a certain direction could exist. Very often, the tested null hypothesis of a ‘zero relationship’ is thus implausible or irrelevant in the first place (Fisher, 1956, p. 42; Johnson, 1999) and has therefore been called a straw‐man hypothesis that serves only to be rejected (Gelman, 2016).

We should not focus only on this straw man, but also discuss test results on alternatives to the null hypothesis of zero effect (Greenland, 2021). Strangely, many if not most researchers present such test results already, but usually do not discuss them—as mentioned above and shown in Figure 2, our traditional 95% confidence intervals show ranges of hypotheses that get *p* > 0.05 when tested using our data.

Furthermore, as indicated by our survey below, we almost never present a formal *a priori* alternative hypothesis and thus cannot claim to test it. Instead, we tend to describe our observed point estimate as though it were a pre‐planned alternative hypothesis, sometimes even calculating retrospective power based on this point estimate, which is useless because it adds no information beyond the obtained *p*‐value (Colegrave & Ruxton, 2003; Greenland, 2012; Hoenig & Heisey, 2001). In practice, with our usual two‐sided tests, the (unstated) alternative hypothesis amounts to ‘anything else but zero’, which in our view does not qualify as a hypothesis at all. There are just too many ways in which a point null hypothesis of zero effect could be false, and rejecting it in favour of ‘anything else’ contributes very little to our knowledge (Szucs & Ioannidis, 2017).

We are deluding ourselves if we believe that the traditional NHST scheme is a meaningful way of evaluating research hypotheses.

## 3 HOW WIDELY ARE THE ABOVE MISCONCEPTIONS RECOGNIZED IN OUR LITERATURE?

To allow a glimpse of the culture of data analysis and the reporting of results in ecology and evolution, we screened 48 papers published in 2020 in the Journal of Evolutionary Biology in the light of the above misconceptions. The 48 empirical articles were randomly selected from the total of 136 articles of the category ‘Research Paper’; if a paper was excluded because it was purely theoretical or used only simulations, a substitute was again drawn at random (more detailed methods are given in Appendix S1, and the screening data are provided on Dryad).

All of the 48 articles adopted the classical NHST framework in which the interpretation of results is based on evaluating *p*‐values against a significance threshold (three studies did not present thresholded *p*‐values but used the equivalent procedure of evaluating whether confidence intervals included zero, and one study used NHST only in the methods section). All studies used the qualifier ‘significant’ or ‘non‐significant’ to rate test results. The significance threshold was apparently always *p* = 0.05, although this was declared explicitly in only 22 of the 48 studies.

Only one study provided a formal description of which null hypothesis was tested, and no single study considered a non‐zero effect size as an informed null hypothesis; hence, all tested hypotheses were the default nulls of ‘zero relationship’. No study specified a formal pre‐planned (or pre‐registered) alternative hypothesis, and accordingly, no study conducted a power analysis before data collection (one study performed post hoc power analysis based on observed parameter estimates, which, as discussed above, is uninformative; Colegrave & Ruxton, 2003; Greenland, 2012; Hoenig & Heisey, 2001). This means that all 48 studies should be considered exploratory (Parker et al., 2016; Szucs & Ioannidis, 2017).

We also counted the number of significance tests reported across the results section of the main body of the paper, including the figures and tables. We considered comparisons of *p*‐values against a significance threshold and checks of whether a confidence interval (CI) contained zero. We also counted all tests that were not made explicit, which usually concerned figures visualizing tests of all treatment groups against each other, while indicating *p*‐values or stars (*) only for the significant comparisons.

In the results sections, the studies reported 49 significance tests on average (median 23, range 0–390). About half of the reported tests were non‐significant (25 on average). These numbers, however, are underestimates because several papers presented many more tests in the Supporting Information, probably particularly non‐significant tests that were not selected for reporting in the main text of the paper. Twelve studies adjusted *p*‐values for multiple testing (using Bonferroni‐type procedures).

Finally, we screened all verbal descriptions of non‐significant tests in the results sections. Among 41 papers that contained such verbal descriptions, 26 (63%) used inappropriate wording for at least one of the tests, implying that non‐significance indicates the absence of an effect (misconception 2); 35 (85%) used adequate wording for at least one of the tests. The most common examples of inadequate wording (‘proofs of the null’) were statements like ‘there was no difference / no effect’ based exclusively on the *p*‐value and not, for example, on an evaluation of all values covered by the CI. We also counted the occasionally occurring ‘no difference was observed’ or ‘patterns were the same’ as inadequate interpretations, since usually effect sizes in a table or figure showed that a difference or correlation *was* observed, and that patterns were *not* the same.

Examples of what we considered appropriate wording were ‘no significant difference’, because a *p*‐value alone does not say anything about the size of an effect (Amrhein et al., 2017) and therefore stating that the difference was non‐significant does not mean that there was ‘no difference’ (although we do not encourage using the significance language). We also considered ‘no difference / effect was found’ and ‘there was no evidence of / no support for’ as appropriate, because such descriptions emphasize absence of evidence and not evidence of absence (Altman & Bland, 1995).

One particularly obvious example of an inappropriate proof of the null was ‘individual estimates were also uncorrelated… (*r* = .258; *p* = .472)’. Another curious example of how the overemphasis on significance tests leads us astray included a table with 102 tests presented only with three‐star notation (***) but no *p*‐values or other test statistics, let alone effect sizes (for references, see the screening data).

One study reported an absurdly small and precise *p*‐value of *p* = 10^−57^, meaning that the probability of observing a relationship at least as large as the one that was found, given that the statistical model and all assumptions like the null hypothesis are correct, is 1/10^57^. This would roughly correspond to the probability of picking a specific atom from our solar system in a random draw. However, it is easy to obtain similarly small *p*‐values by ‘testing’ a statistical model very far from the data that we observe in our study; for example, a few data points close to the diagonal of y = x suffice to conclude that the default hypothesis of a zero correlation is extremely incompatible with our data. A very small *p*‐value therefore does often not mean that the study found ‘very strong evidence for the effect’ (as researchers usually claim); but it shows that the model and null hypothesis chosen for testing are too far away from reality to be useful and that we should come up with a better model.

Based on our screening, we conclude that the research protocol described above under the heading *What are the problems?* is by no means a caricature, but a relatively accurate portrait of how studies in evolutionary biology are at present conducted and reported. The vast majority of investigations in our field still follow the traditional NHST scheme, despite ample exposure of its problems for about a century (Amrhein et al., 2017; Berkson, 1938; Boring, 1919; Gigerenzer, 2018; Greenland, 2017; Hurlbert & Lombardi, 2009; Johnson, 1999; Mayo, 2018; McShane et al., 2019; Oakes, 1986; Rozeboom, 1960; Szucs & Ioannidis, 2017; Yates, 1951; Ziliak & McCloskey, 2008), and despite broad agreement within the community of statisticians that the current state of NHST usage is damaging to science (Amrhein et al., 2019a; Benjamin et al., 2018; Seibold et al., 2021; Wasserstein & Lazar, 2016; Wasserstein et al., 2019).

We are forced to recognize that the problems related to NHST abound in our literature: overconfident claims about ‘discovered effects’ and overestimated effect sizes for significant tests, and a great proportion of erroneously dismissed but potentially biologically relevant effects and underestimated effect sizes for non‐significant tests. Clearly, it is high time for improving our conventions of data analysis and reporting of results.

## 4 MOVING FROM SIGNIFICANCE TESTING TO ESTIMATION AND COMPATIBILITY

Undoubtedly, science is progressing despite the problems with NHST highlighted above. One main reason is that although initial studies on a given phenomenon often suffer from biases such as inflated effect sizes introduced by the significance filter, these biases are often reduced in replication studies (effect sizes in replications are usually smaller than in the original studies; Brembs et al., 2013; Errington et al., 2021; Jennions & Møller, 2002; Open Science Collaboration, 2015). With every replication study that contributes new data in a relatively unbiased way, the substrate for building and refining models about principles in nature becomes more solid. Recognizing that this accumulation of, and synthesis across, data sets lies at the heart of the scientific progress (Glass, 2010; Nichols et al., 2019, 2021) has several conceptual and methodological implications.

### 4.1 Study questions rather than hypotheses

We no longer need to test hypotheses framed as ‘there is a relationship’, a generalized claim for which single studies are usually unable to give sufficient support. The contribution of single studies to science is the estimation of the direction and strength of potential relationships and of their uncertainty, based on observations and experiments. More generalized scientific conclusions will typically require information to be combined across multiple studies, each performed under their own set of conditions and assumptions and hence describing unique patterns and variation. Such meta‐analyses summarize data or effect estimates and their precision, not the number of claims about hypotheses such as ‘we confirmed there is a relationship (*p* < 0.05)’ (Gurevitch et al., 2018; Halsey, 2019).

It seems that in ecology and evolutionary biology, we are generally driven by curiosity, broad study questions and multi‐factorial hypotheses rather than by clear‐cut, isolated hypotheses that can be either ‘rejected’ or ‘accepted’ (Glass, 2010; Nichols et al., 2019). We should therefore primarily report descriptions of relationships and their uncertainty, and refrain from perceiving our NHST studies as confirmatory. Without pre‐planned (and pre‐registered) quantitative predictions and justified decision rules, our studies are exploratory (Parker et al., 2016; Szucs & Ioannidis, 2017), whatever statistical framework we use for analysis.

There is no shame in admitting that our research, for example in the 48 studies that we screened, is generally exploratory and guided by broad questions rather than by narrow hypotheses. But if we cannot really provide yes‐or‐no answers, it also makes no sense to force students and study authors to formulate the usual array of dichotomized hypotheses in the introductions of their papers. Instead, it makes more sense to ask ‘how strong is the relationship?’ or ‘is it strong enough to matter?’, and to formulate our expectations about the direction and size of that relationship.

### 4.2 Full reporting rather than filtering of results

We should abandon filtering our study outcomes based on statistical significance, no matter what significance threshold is used (Amrhein & Greenland, 2018; Benjamin et al., 2018). If our research is well conceptualized and properly carried out, any emerging result is a useful contribution to science, deserves discussion within the focal research context and deserves publication. In this light, there is also no problem in analysing one and the same data set in different ways to explore the sensitivity of results to violations of assumptions (Greenland, 2021)—as long as these explorations are fully reported, not just the ones producing a desired outcome such as the smallest *p*‐value. And perhaps even more important than full reporting of summary statistics is that we ensure free access to the underlying raw data for meta‐analysts (Lawrence et al., 2021; Whitlock et al., 2010).

Giving up the practice of favouring statistically significant over non‐significant effects, or of applying similar filtering methods based, for example, on Bayes factors or the Akaike information criterion (AIC), will naturally reduce the upwards bias of reported effect sizes. For this and other reasons, there is now a broad movement of statisticians and researchers advocating that the labels ‘statistically significant’ or ‘non‐significant’, and analogous decorations of *p*‐values such as stars or letters, should have no place in research articles (Amrhein et al., 2019a; Hurlbert et al., 2019; Lakens et al., 2018; Trafimow et al., 2018; Wasserstein et al., 2019).

### 4.3 Compatibility rather than confidence

Finally, the overconfidence resulting from NHST should give way to a greater acceptance of uncertainty and embracing of variation (Gelman, 2016). Our data and statistics are generally more noisy and biased than we recognize. Appreciating that a single study will rarely suffice to establish a robust model of a biological principle will remove the pressure to oversell potential effects, or even to turn tests statistically significant by more or less subtle data manipulation (Fraser et al., 2018; Gelman & Loken, 2014).

Because the main value of our research is the estimation of effect sizes and of their uncertainty, our emphasis should shift to the clear and comprehensive presentation of point estimates and their associated interval estimates. A straightforward way of doing so is interpreting the classical confidence intervals as compatibility intervals (Amrhein et al., 2019a, b; Gelman & Greenland, 2019; McElreath, 2020; Rafi & Greenland, 2020).

For instance, results could be summarized as follows: ‘In our study, the average weight increase was 7.5 g; possible values for the true average weight increase that were most compatible with our data, given our statistical model, ranged from 2.0 to 13.1 g (95% CI)’. This clearly conveys more insight than ‘We found a significant average weight increase of 7.5 g (*p* = 0.009)’.

If the interval includes effect sizes in the opposite direction, we could write: ‘In our study, the average weight increase was 5.0 g; possible values for the true average weight change that were most compatible with our data, given our statistical model, ranged from a 1.5 g decrease to an 11.5 g increase (95% CI)’. Compare this with the vacuous statement ‘We found a non‐significant average weight increase of 5.0 g (*p* = 0.13)’.

In both cases, the researchers should then discuss the biological implications of a possible weight change across the entire observed intervals.

From a traditional hypothesis testing perspective, the 95% CI shows the values ‘most compatible with our data’ because it covers all null hypotheses that would get *p* > 0.05 when tested using our data. As just exemplified, a strength of compatibility intervals is to direct our attention to a range of most compatible true effect sizes (hypotheses) in the light of our data and our statistical model. However, compatibility intervals should not be misused for dichotomous judgments based on whether or not they overlap an effect size of zero, as this shares all the problems inherent in traditional NHST. Of course, our plea for interval‐based statistical inference extends to compatibility intervals obtained, for example, using Bayesian methods (traditionally called ‘credible intervals’; McElreath, 2020) or resampling procedures (Manly & Navarro Alberto, 2020).

An even stronger option for compatibility‐based inference that avoids the arbitrary thresholds at which the lines of intervals must end is the compatibility curve (Infanger & Schmidt‐Trucksäss, 2019; Poole, 1987; Rafi & Greenland, 2020). This underused tool allows evaluating the most compatible effect sizes in the light of the data and the statistical model, exposing two important elements hidden in conventional intervals: that compatibility does not stop where an interval would end, but extends beyond it; and that the compatibility of effect size estimates (hypotheses) is not uniform across an interval, but declines as we move away from the point estimate. We provide examples of compatibility curves applied to simulated regressions in Figure 2 and Appendix S1, and a working protocol based on bootstrap resampling as well as on conventional parametric ‘confidence’ intervals in our code compilation.

## 5 CLOSING REMARKS

Our call to give up NHST in favour of compatibility‐based inference is not a call to completely abandon *p*‐values or *p*‐value thresholds: again, a conventional 95% compatibility interval displays hypotheses that are not rejected because they get *p *> 0.05, and compatibility curves also visualize *p*‐values (Figure 2). Our main point is that when interpreting intervals, the focus is on many hypotheses rather than on just the one of zero effect, and on uncertainty rather than on categorical statements about whether an effect has been ‘demonstrated’ or not.

Of course, many other options exist for effectively describing and communicating effect estimates and their uncertainty (Colquhoun, 2014; Cumming, 2014; Gurevitch et al., 2018; Korner‐Nievergelt et al., 2015; McElreath, 2020; Nakagawa & Cuthill, 2007; Rafi & Greenland, 2020). Our task for the future is to exploit and to teach these options creatively, keeping in mind that all approaches have their strengths and weaknesses and answer slightly different questions, and that probably none of them is universally applicable or necessarily superior (Gigerenzer & Marewski, 2015; Goodman, 2016). What we hope to have made clear with this note, however, is that we can safely give up null hypothesis significance testing and the reporting of ‘statistical significance’. Doing so will help overcome problems with which science has struggled for decades.

## CONFLICT OF INTEREST

The authors have no conflict of interest to declare.

## AUTHOR CONTRIBUTIONS

Coding was done by DB. All other work was conducted by both authors in similar parts.

### PEER REVIEW

The peer review history for this article is available at https://publons.com/publon/10.1111/jeb.14009.

## Supporting information

Supplementary Material

## Data Availability

All code used for simulations and plotting as well as the scoring sheet summarizing our literature screen are available from the Dryad digital repository (https://doi.org/10.5061/dryad.zkh1893c8).
