# Supplementary material for: Why and how we should join the shift from significance testing to estimation
Source: J Evol Biol. 2022 May 18;35(6):777–87. doi: 10.1111/jeb.14009 (PMC9322409; doi:10.1111/jeb.14009)
Supplement: Supplementary file 1 — Supplementary Material [file JEB-35-777-s001.pdf]

Appendix S1 to

Why and how we should join the shift from significance testing to estimation

by Daniel Berner & Valentin Amrhein

Simulations

To illustrate conceptual issues in NHST, we used the R language (R Core Team, 2020) to simulate data sets of two correlated variables (x = predictor; y = response). The predictor was drawn at random from a normal distribution with a mean of zero and a standard deviation of 0.5. The response was constructed by assuming the positive linear relationship $y = 0.5x$. To make the association between the variables noisy, we then added to each element of y a random draw from a normal distribution with a mean of zero and a standard deviation of 0.5 (scenario (a) with stronger correlation), or a standard deviation of 1 (scenario (b) with weaker correlation). The exact correlations between x and y obtained in this way were 0.447 and 0.243, respectively, as determined empirically based on a sample size of n = 50 million.

For sample sizes ranging from eight to 100 in steps of two, we generated 10,000 such bivariate data sets under both simulation scenarios. For each data set, we then analyzed the correlation between x and y by using the *cor.test* function, and saved the correlation coefficient (i.e., the effect size) and the associated *P*-value for the default null hypothesis of zero correlation. This allowed us to characterize the *P*-value distribution for each sample size based on the 5 and 95 as well as the 25 and 75 percentiles (i.e., the 90% and 50% intervals around the median). Classifying the correlation tests as 'statistically significant' ($p < 0.05$) or 'non-significant' ($p > 0.05$), we determined the proportion of significant tests. We then characterized the distribution of the effect size estimates (median, and 90% and 50% intervals around the median) separately for the significant and non-significant tests. The R code used for data simulation, analysis and graphing is available from the Dryad repository (https://doi.org/10.5061/dryad.zkh1893c8).

Literature review

We randomly chose four articles from each of the 12 issues of the Journal of Evolutionary Biology published in the year 2020 (volume 33). We considered the article category 'Research Papers' only, and we ignored purely theoretical studies (e.g., pure simulation studies). The 48 papers were examined for whether statistical inference involved dichotomous conclusions based on the evaluation of *P*-values from statistical tests against a significance threshold, or based on whether confidence intervals overlapped zero. We assessed whether test results were reported by using the qualifier '(non-)significant', what significance threshold (alpha level) a study adopted, and whether this threshold was declared explicitly in the paper. We also determined the number of significance tests presented, focusing on the results section of the main article only, including the tables and figures; additional testing presented in the methods section or the Supporting Information was ignored for the counts. We counted all mentions of a *P*-value, even if a *P*-value was

reported both in the text and in a table or figure, because sometimes *P*-values were interpreted twice and differently in a figure legend and the main text. For post-hoc tests reported by using stars or letter coding in figures or tables, each contrast represented a significance test and was counted as such. We further examined if authors pre-specified null hypotheses (that could be different from an effect size of zero), and whether investigations were tailored to biologically informed alternative hypotheses. The latter would have involved defining effect sizes of interest prior to data collection (based on the literature or pilot studies), and estimating what sample sizes would be needed to reject false null hypotheses with a desired probability (i.e., power). As studies typically reported numerous significance tests, we additionally examined if attempts were made to adjust for multiple testing by searching for the key words “multiple testing”, “Bonferroni”, and “false discovery rate”. Finally, we screened the articles for inappropriate conclusions that there was no effect because the effect was statistically non-significant. The scoring sheet summarizing our literature screening and giving more explanations is provided on Dryad.

Compatibility curves

Using the simulation model (a) from above (i.e., the stronger correlation), we generated two exemplary sample data sets, one with n = 30 and one with n = 80. The expected (true) correlation was the same in both cases (r = 0.45). To construct non-parametric compatibility curves, we bootstrapped (resampling with replacement) each sample 100,000 times, each time calculating and recording the coefficient of the correlation between x and y. From the two distributions thus obtained, we then determined the lower and upper limit of the 0-99% compatibility (“confidence”) intervals (step size: 1%), as defined by symmetric (i.e., two-tailed) percentiles. For example, the 99% compatibility interval (bottom of the curve) was delimited by the bootstrapped correlation coefficients located at 0.5% and 99.5% of their ordered distribution, while the 0% compatibility interval (peak of the curve, or point estimate) represented the median of this distribution. Finally, we plotted the two endpoints of the compatibility intervals against their compatibility levels, thus obtaining the compatibility curves.

We also carried out an analogous parametric analysis. For this, we applied the *cor.test* function to the two simulated data sets, sequentially raising the value of the compatibility level (*conf.level* argument) from 0-99% in steps of 1%. Recording the upper and lower limits of the compatibility intervals obtained in this way again allowed us to draw compatibility curves. Because the non-parametric approach makes fewer assumptions regarding the distribution of the data, we present only the non-parametric compatibility curves in the main text. However, both approaches are graphed together in Figure S1 below. The R code used for producing compatibility curves, both non-parametrically and parametrically, is shared on Dryad.

Figure S1

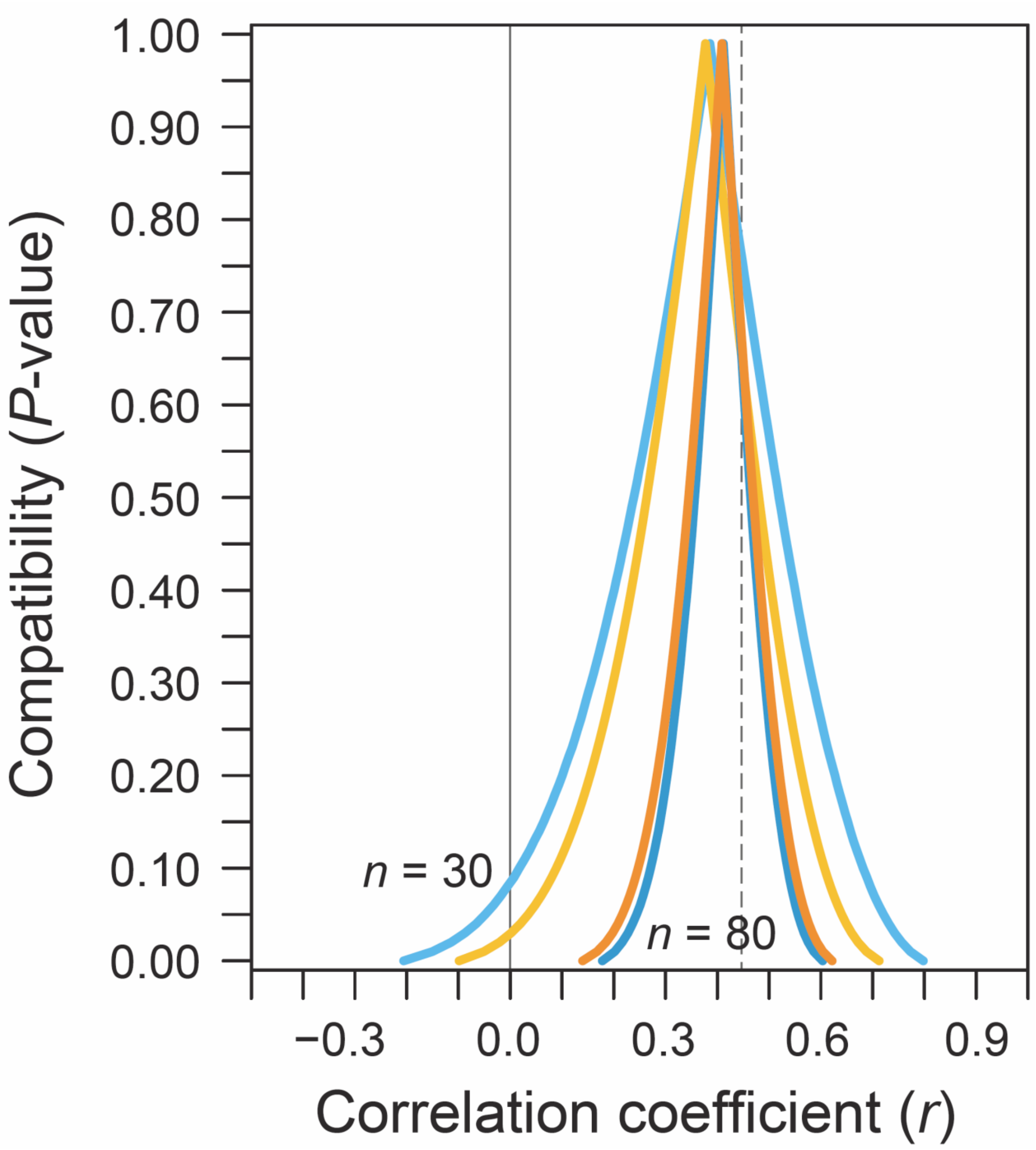

Figure S1 Parametric and non-parametric compatibility curves. The data generation protocol and the graphing conventions are identical to Figure 2, except that the parametric compatibility curves for n = 30 (light orange) and n = 80 (dark orange) are superposed on their non-parametric (bootstrap-based) counterparts (blue). The locations and shapes of the curves differ from those in Figure 2 because a new random data set was drawn for each sample size. Note that depending on the specific variational properties of a given sample, the two types of compatibility curves may be nearly congruent, or one or the other type may be wider.
